# A Label-Free and Antibody-Free Molecularly Imprinted Polymer-Based Impedimetric Sensor for NSCLC-Cells-Derived Exosomes Detection

**DOI:** 10.3390/bios13060647

**Published:** 2023-06-13

**Authors:** Jingbo Zhang, Quancheng Chen, Xuemin Gao, Qian Lin, Ziqin Suo, Di Wu, Xijie Wu, Qing Chen

**Affiliations:** 1 School of Pharmaceutical Science, Xiamen University, 4221 Xiang’an South Road, Xiamen 361102, China; jingbozh@stu.xmu.edu.cn (J.Z.); chenqc@xmu.edu.cn (Q.C.); holygxm@xmu.edu.cn (X.G.); 32320211154294@stu.xmu.edu.cn (Q.L.); 32320221154411@stu.xmu.edu.cn (Z.S.); amoywd@stu.xmu.edu.cn (D.W.); 2 Department of Cardiac Surgery, Xiamen Cardiovascular Hospital, Xiamen University, 2999 Jinshan Road, Xiamen 361010, China

**Keywords:** exosomes, molecular imprinted polymers (MIPs), antibody-free, impedimetric sensor

## Abstract

In this study, a label-free and antibody-free impedimetric biosensor based on molecularly imprinting technology for exosomes derived from non-small-cell lung cancer (NSCLC) cells was established. Involved preparation parameters were systematically investigated. In this design, with template exosomes anchored on a glassy carbon electrode (GCE) by decorated cholesterol molecules, the subsequent electro-polymerization of APBA and elution procedure afforded a selective adsorption membrane for template A549 exosomes. The adsorption of exosomes caused a rise in the impedance of the sensor, so the concentration of template exosomes can be quantified by monitoring the impedance of GCEs. Each procedure in the establishment of the sensor was monitored with a corresponding method. Methodological verification showed great sensitivity and selectivity of this method with an LOD = 2.03 × 10^3^ and an LOQ = 4.10 × 10^4^ particles/mL. By introducing normal cells and other cancer cells derived exosomes as interference, high selectivity was proved. Accuracy and precision were measured, with an obtained average recovery ratio of 100.76% and a resulting RSD of 1.86%. Additionally, the sensors’ performance was retained at 4 °C for a week or after undergoing elution and re-adsorption cycles seven times. In summary, the sensor is competitive for clinical translational application and improving the prognosis and survival for NSCLC patients.

## 1. Introduction

Lung cancer is currently regarded as the leading cause of cancer-related death worldwide. As NSCLC accounts for about 80% of cases and shows a 5-year survival rate as low as 10–15% [1,2], early diagnosis plays an important role in improving the prognosis and survival [3].

Exosomes are extracellular vesicles with a diameter of about 40–150 nm originating from endosomes generated in most cells [4]. Endowed with fairly good stability and accumulation in the circulatory system, these membrane-enclosed vesicles can be found in body fluids (e.g., blood, urine and cerebrospinal fluid) and secretions (e.g., tears, semen, sweat) [5]. Thus, exosomes are considered to be suitable clinical biomarkers for early cancer diagnosis due to the abundant packaging of biomarkers in their mother cell [6]. Similar NSCLC-derived exosomes from tumor cells (e.g., A549, H460 and H1299) carry different expression levels of a range of proteins (e.g., epithelial cell adhesion molecule (EpCAM and carcinoembryonic antigen (CEA)), which results in distinct surface phenotypes reflecting the cancer occurrence and progression [7].

Current detection methods for exosomes commonly start with the specific binding of the signal label and exosomes via antigen–antibody interaction [8,9,10]. However, the intrinsic fragility of proteins makes them vulnerable to environmental disturbances such as heat, acid, alkaline and organic solvents, so the robustness of the method and the storage life of the sensor are hampered [11]. Furthermore, it takes complex Western blot or other immunology tests to specify the characteristic protein and design the signal label of target exosomes [12].

To overcome the obstacle of recognizing constituents and to minimize the cost, molecularly imprinted polymers (MIPs) have drawn much attention thanks to their capacity as artificial antibodies with a better performance in stability, more predictability in structure and an easier preparation process [13]. Based on covalent and non-covalent bonds between functional monomers and templates, the polymerization proceeds around templates. Under steric hindrance, cavities highly complementary to the templates in spatial shape and chemical groups can be built up after the elution of the template. Once a new sample is added on to the MIPs, these cavities adsorb similar molecules or particles in the sample with high selectivity for size, shape and chemical group arrangement. Up till now, templates fitting this technology have ranged from small molecules and ions to biomacromolecules, viruses, bacteria [14] and even tumor cells [15].

Among numerous chemical groups with the capability to bind the templates specifically, the boronic acid group can interact with various protein targets including hydroxyl, *cis*-dihydroxyl, metal ions, etc., in versatile docking types [16]. Therefore, functional monomers containing boronic acid groups can well recognize the intensive O atoms of protein, saccharide, glycosides and other biomolecules [17]. Making use of boronic acid affinity, aminophenylboronic acid (APBA) was found to be a promising functional monomer for biomolecules such as glycoproteins [18] and sialic acid [19].

Furthermore, the ultimate quantification method is typically obtained by converting the captured exosome count into a directly measurable signal such as ultraviolet and visible absorption, fluorescence, surface plasma resonance, electric current, impedance, etc. [20,21,22]. Comparatively, electrochemical impedance can be obtained faster and easier with indicating information about the sensors’ interface [23].

Therefore, combining boronic acid affinity as the recognition component, a simple exosomes-imprinted polymer (EIP) biosensor for A549-cells-derived exosomes was designed. It is easy to quantify the exosomes’ concentration using electrochemical impedance spectroscopy (EIS), owing to the intrinsic poor conductivity of exosomes. A method validation was also conducted in this study.

## 2. Materials and Methods

### 2.1. Reagents and Apparatus

A549-cells-derived exosomes were separated and purified by Lifeint (Xiamen, China). Cholesteryl chloroformate (98%), *ortho*-aniline boronic acid (APBA, 98%), phosphate buffer solution (PBS, 10 × pH = 5, 1 × pH = 7.4), carbonate buffer solution (CBS, 0.5 M, pH = 10) and sodium hydroxide (95%) were purchased from Macklin (Shanghai, China). Hydrochloric acid (36%) was purchased from Sinopharm Chemical Reagent (Shanghai, China). Potassium ferricyanide, potassium ferrocyanide and potassium chloride (AR) were purchased from Xilong Scientific (Shantou, China). Sodium fluoride (99%) was purchased from Innochem (Beijing, China). Tetrahydrofuran (THF, AR) was purchased from Energy Chemical (Shanghai, China). Triton X-100 (BR) was purchased from Aladdin Biochemical Technology (Shanghai, China). DMEM high glucose culture media was purchased from Cytiva (Marlborough, MA, USA). Penicillin–streptomycin was purchased from Gibco (Waltham, MA, USA). Exosome-depleted FBS Media Supplement was purchased from SBI (Palo Alto, CA, USA). Ultrapure water was purified using Milli-Q^®^ Advantage (Burlington, MA, USA).

Electrochemical measurements were performed with a CHI660E electrochemical workstation (CH Instruments, Austin, TX, USA). The morphology of purified exosomes was examined using an HT-7700 (Hitachi, Tokyo, Japan) transmission electron microscope (TEM). The particle size distribution of purified exosomes and corresponding quantification results were obtained with N30E (NanoFCM Inc., Xiamen, China) Nanoparticle Flow Cytometry. The morphology and elemental composition of samples were examined using a SUPRA55 (Carl Zeiss Microscopy, Oberkochen, Germany) scanning electron microscope (SEM) with an energy dispersive spectrometer (EDS). Fourier transform infrared spectroscopy (FTIR) was conducted using a Nicolet iS10 spectrometer (Thermo Scientific, Waltham, MA, USA) with a Smart iTR Attenuated Total Reflectance (ATR) Sampling Accessory.

### 2.2. Cell Culture

Human non-small-cell lung cancer cells (A549 cells) were chosen as the tumor cell model, with human lung epithelial cells (BEAS-2B cells) chosen as the normal cell model, stage IV human breast cancer cells (4T1 cells) for the animal model, and human cervical cancer cells (Hela cells) chosen as interferences. All kinds of cells were incubated with DMEM medium containing 10% FBS and 1% antibiotic (streptomycin and penicillin) and were placed in an incubator containing 5% CO_2_ at 37 °C.

### 2.3. Exosome Separation and Purification

Cultured A549 cells were rinsed with 1× PBS when 60–70% of the space of the culture dish was taken up. Subsequently, cells were incubated with 10% exosome-depleted fetal bovine serum for 48 h with the other conditions unchanged, as is narrated in Section 2.2. Then the supernatant was collected and centrifugated at 4 °C with 2000× *g* for 30 min to remove the debris of the cells.

After the removal of sediment, the newly obtained supernatant was centrifugated again at 4 °C with 10,000× *g* for another 45 min to eliminate larger extracellular vesicles such as microvesicles, apoptotic bodies and large oncosomes. Afterwards, the supernatant was filtered with a 0.45 μm filter membrane, followed by transferring the filtrate to a new centrifuge tube and centrifugation at 4 °C with 100,000× *g* for 70 min.

Finally, the obtained sediment was redispersed with 1× PBS at 4 °C and the former 100,000× *g* centrifugation process was performed again with the redispersion of the freshly obtained sediment in 1× PBS at 4 °C.

The prepared exosomes were stored at −80 °C for further use.

### 2.4. Electrode Modification

All used GCEs were held vertically and polished with alumina powder (Al_2_O_3_, 1.0, 0.3 and 0.05 μm in turn) in a ∞ route to a mirror finish. Then they were washed in deionized water ultrasonically for 3 min. The cleaned electrodes were dried under infrared lamps. In the 1× PBS (pH = 7.4, 0.01 M) containing 10 mM [Fe(CN)_6_]^3−/4−^ (1:1) and 0.1 M KCl, cyclic voltammetry (CV) was recorded with a scanning rate of 50 mV/s in the voltage window −0.2–0.6 V (vs. Ag/AgCl) to test if electrodes were thoroughly cleaned. The potential difference between the two redox peaks should be smaller than 80 mV and as close to 64 mV as possible.

The polished GCEs were activated in 1M NaOH solution via scanning 10 rounds in the voltage range of −0.1–1.2 V (vs. Ag/AgCl) with a speed of 50 mV/s in CV mode so that hydroxyl and carboxyl groups were introduced to the surface of GCEs.

Finally, so-coped GCEs were immersed into THF containing 25 mM cholesteryl chloroformate for 30 min to make electrodes that were modified with cholesteryl groups. It is also worth noting that involved electrodes should be carefully rinsed with deionized water after each step.

### 2.5. Exosome Fixation and Electrochemical Polymerization of EIP Membrane

To fix template exosomes onto working GCEs, cholesteryl-modified electrodes were immersed into a 1× PBS suspension of A549-derived exosomes with a concentration of 2 × 10^7^ particles/mL for 15 min.

Afterwards, an EIP membrane was afforded in a three-electrode cell with a modified GCE as the working electrode, an Ag/AgCl (in 3 M KCl) reference electrode and a platinum wire counter electrode. CV was performed from −0.1 V to 1.1 V for 10 cycles at a scan rate of 50 mV/s and held at 0.8 V for 15 s in a 10× PBS with 40 mM 3-APBA and 300 mM NaF (catalyzing the polymerization of APBA [24]) as an electrolyte to deposit F^-^-doped poly-APBA (p-APBA) around the template exosomes. Eventually, the EIP membrane was obtained after the elution of templates with 10 vt% Triton X-100–0.05 M CBS solution; meanwhile, the biosensor was established. The non-imprinting polymers (NIP) membrane was prepared using a similar method without template exosomes fixed onto modified GCEs.

### 2.6. The EIP-Based Impedimetric Sensor

For the target exosomes’ re-adsorption, the 10 μL dispersion of them in 1× PBS was dropped onto the surface of the sensor and incubated for 10 min. The impedance of the sensor was measured using EIS in 1× PBS (pH = 7.4) containing 10 mM [Fe(CN)_6_]^3−/4−^ (1:1) and 0.1 M KCl before and after re-adsorption (marked as R_0_ and R) under the open circuit potential as the initial potential, frequency from 1 × 10^5^–1 × 10^−2^ Hz and amplitude at 5 mV. The relative difference (ΔR_r_) of R_0_ and R (calculated according to Formula (1)) indicated the concentration of template exosomes in the sample.

ΔR_r_ = (R − R_0_)/R_0_, (1)

All impedance data obtained were fitted using ZSimpwin (version 3.60) to get the specific value of impedance on the sensor surface.

## 3. Results and Discussion

### 3.1. Design of the EIP-Based Impedimetric Sensor

**Scheme 1:**
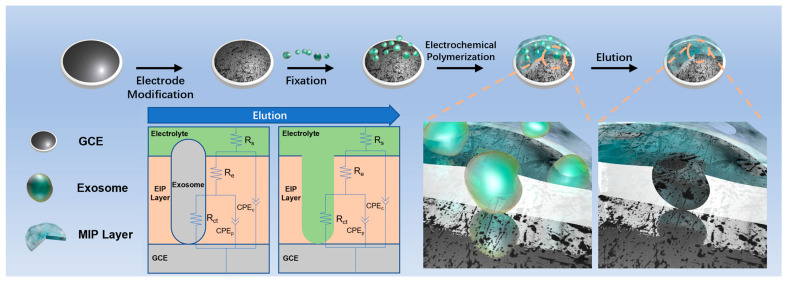
Schematic illustration of the preparation of the EIP biosensor and the equivalent circuit corresponding to the element in sensing process.

In Scheme 1, the preparation and structural details of the sensor are shown. First of all, introduced template exosomes are anchored onto the modified GCE surface via the affinity between cholesterol and the phospholipid bilayer of exosomes [25]. Consequently, the later polymerization of APBA forms the foundation of the EIP layer. By introducing the CBS solution of Triton X-100 as an eluent, the solubility of exosomes is increased with the hydrophobic end of Triton X-100 inserted into the phospholipid bilayer, and the alkaline solution ensures that the exosomes’ surface protein reversibly binds to the boronic acid groups of the EIP layer. Eventually, the elution of template exosomes leaves plenty of cavities with condensed F^-^-bonded boronic acid groups which are thoroughly complementary to the template exosomes. Afterwards, the EIP biosensor is established.

As for the sensing process, the multi-layered structure of the sensor is always demonstrated as the equivalent circuit shown. Five elements reflecting electrolyte resistance (R_s_), the capacitance of the polymer coating layer (constant phase element, CPE_c_), the resistance of the coating layer (R_e_), the charge transfer resistance in the imprinting cavities (R_ct_) and the capacitance of the electrical double layer (CPE_p_) are suitable to describe the feature of the sensor [26]. As a consequence of the poor conductivity of exosomes, R_ct_ increases significantly after the re-adsorption of imprinted exosomes in the EIP cavities.

**Scheme 2:**
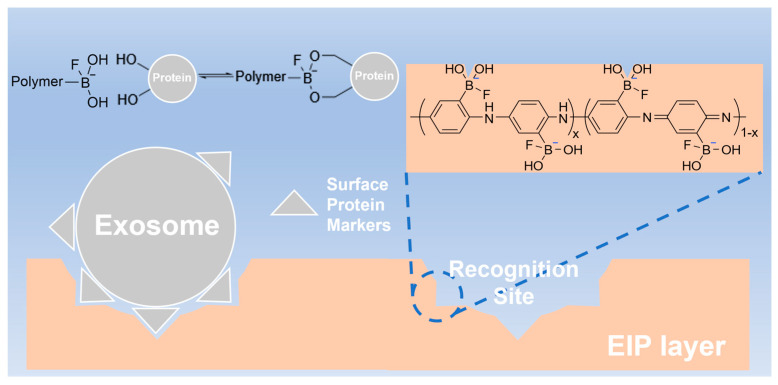
Detailed interaction between target exosomes and EIP layer.

The detailed interaction between target exosomes and the EIP layer is illustrated in Scheme 2. The elution of template exosomes leaves abundant pits with a specific size and shape as recognition sites of the sensor. As a result of polymerization spatially hindered by anchored template exosomes on the surface of the modified electrode, such afforded pits are highly complementary to anchored exosomes in both shape and size. Moreover, APBA molecules are allocated in a complementary pattern to the chemical groups on template exosomes by the boronic affinity between the APBA and surface proteins, saccharides as well as glycosides.

Consequently, the correspondence of recognition sites and template exosomes spatially and chemically endow sites with the capacity to specifically adsorb particles with not only a similar size and shape but similar surface proteins, saccharides, glycoside types and allocation as well in analytes. Therefore, exosomes are distinguished from other species of extracellular vesicles such as microvesicles (about 100–1000 nm in diameter), apoptotic bodies (about 1000–5000 nm in diameter) and large oncosomes (about 1–10 μm in diameter) [27].

Hence, the sensor can be employed for simple, sensitive and quick detection of target exosomes’ concentration.

### 3.2. Characterization of Purified Exosomes

**Figure 1:**
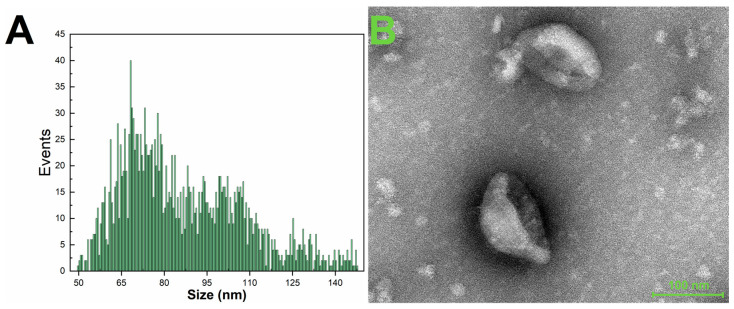
(**A**) Particle size distribution of purified exosomes, the number of exosomes with different diameter is counted; (**B**) 60,000× TEM image of purified exosomes.

The particle size distribution of purified A549-derived exosomes was measured with NanoFCM. The size of exosomes ranged from 49.75 nm to 147.75 nm in diameter (Figure 1A), which identified the afforded sediment as wanted exosomes. Correspondingly, the concentration of the final dispersion of exosomes was calculated to be 2.03 × 10^10^ particles/mL.

Moreover, the morphology of purified exosomes was characterized using TEM. The obtained TEM image (Figure 1B) exhibits the typical cup-shaped appearance with a size in accordance with the NanoFCM results. Therefore, such purified exosomes were qualified to serve as templates for imprinted polymerization.

### 3.3. Morphology of the EIP Membrane

**Figure 2:**
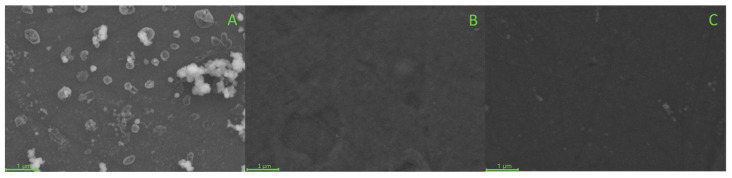
(**A**) 15,000× SEM image of EIP membrane before elution; (**B**) 15,000× SEM image of EIP membrane after elution; (**C**) 15,000× SEM image of NIP membrane after elution.

The surface morphology of the sensor with the EIP membrane was investigated using SEM. The image shows that the template exosome particles dispersed homogeneously on the membrane (Figure 2A), and the following elution step so completely removed all exosomes that pits complementary to the template exosomes (Figure 2B) on both spatial and chemical aspects were afforded. Yet, such pits are not observed on the image of the NIP membrane (Figure 2C). The obtained NIP membrane exhibited a smoother morphology than the EIP, as the polymerization was carried out equally on the surface of the modified GCE without the hindrance of anchored exosomes. Therefore, it can be deduced that the membrane’s morphology was highly dependent on template exosomes.

**Figure 3:**
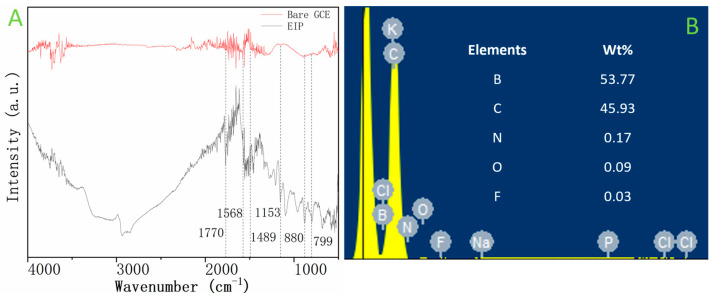
(**A**) ATR–FTIR spectrum of bare GCE and EIP membrane, with dotted line to locate the peak; (**B**) EDS spectrum (the intensity of yellow peaks representing the content of elements labelled) of EIP membrane and corresponding elements weight percentage.

Furthermore, the generation of p-APBA can be confirmed by the emergence of corresponding peaks (ν_C=N_, 1700 cm^−1^; ν_C=C_, 1568 and 1489 cm^−1^; ν_C-N_, 1153 cm^−1^; δ_C-H_, 880 and 799 cm^−1^) on the FTIR spectrum (Figure 3A). It is also proved by the EDS spectrum (Figure 3B) of the membrane showing the qualitative identification of the involved elements of p-APBA.

### 3.4. Methodology Validation of the Impedimetric Sensor

**Figure 4:**
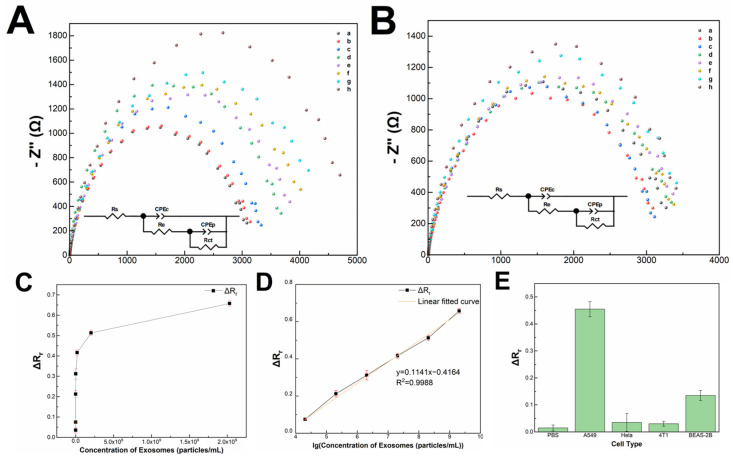
(**A**) Impedance data obtained after re-adsorption in PBS dispersion of A549-derived exosomes with the concentration of 0, 2.03 × 10^3^, 2.03 × 10^4^, 2.03 × 10^5^, 2.03 × 10^6^, 2.03 × 10^7^, 2.03 × 10^8^ and 2.03 × 10^9^ particles/mL on EIP sensor, displayed in Nyquist plot (a–h); (**B**) series Nyquist plot obtained after the re-adsorption process with the same dispersion as (**A**) on NIP sensor; (**C**) calibration curve of impedance response and concentration of exosomes in the same range; (**D**) linear relationship between impedance response and the logarithm of concentrations of exosomes in the same range. (**E**) Relative change of impedance before and after re-adsorption in PBS and culture media of different cell lines incubated for 48 h (*n* = 3).

Under optimal conditions (the optimization process can be found in Supplementary Material Section S1 and the discussion can be found in Section S2), the EIP-based impedimetric sensor’s performance in detecting variable concentrations of the A549-derived exosomes was evaluated. One of the prepared EIP and NIP sensors for each was tested with a series dispersion of A549-cells-derived exosomes. The corresponding impedance data are shown in a Nyquist plot (Figure 4A). The shrinkage of the semi-circle indicated that the R_ct_ of the EIP sensor decreased along with the decreasing concentration of exosomes from 2.03 × 10^9^ to 2.03 × 10^3^ particles/mL, while the same trend is not observed on the plot of the sensor loaded with NIP membranes (Figure 4B). As illustrated in Figure 4C,D, the impedance response increased correspondingly with the concentration of exosomes. A linear relationship between the impedance response and the logarithmic value of the exosome concentrations from 2.03 × 10^9^ to 2.03 × 10^4^ particles/mL can be established. The obtained calibration curves fit the linear Equation (2)

ΔR_r_ = 0.1141 lgc − 0.4164, (2)

with a correlation coefficient of 0.9988.

The specificity and performance of the sensor on biological samples were also evaluated. The sensor was tested with A549 and several other cell lines’ culture media, such as Hela, 4T1, and BEAS-2B cells, incubated under the same conditions described in Section 2.2 for 48 h. As shown in Figure 4E, no significant signal is observed in the culture media of Hela and 4T1 cells, and only a relatively low response is observed in the sample from BEAS-2B cells, suggesting that this sensor has great selectivity for A549-derived exosomes. Moreover, the LOD and LOQ of the method were determined as 2.03 × 10^3^ and 4.10 × 10^4^ particles/mL, respectively, based on three and ten times the standard deviation of the signal obtained in 1× PBS solution (Figure 4E) as method noise.

The selective impedance response is owing to the selective adsorption ability of the EIP membrane. In the culture media, particles with the largest size such as cells and their debris can be excluded by simply centrifuging with 2000× *g*, after which the supernatant is only composed of small extracellular vesicles generated from different cell lines. Then, vesicles with just the same size, shape and surface biomacromolecule arrangement, i.e., A549-cells-derived exosomes are adsorbed into the recognition sites on the EIP membrane via the re-adsorption incubation step. Thus, other interfering extracellular vesicles such as microvesicles, apoptotic bodies and large oncosomes are expelled because of the rise of the impedance.

In conclusion, the different response with various cell lines reflects the intrinsic distinction of each kind of exosome. It is implied from the relatively larger signal obtained with the culture media of BEAS-2B cells that A549- and BEAS-2B-cells-derived exosomes have more spatial and chemical features in common than A549 and other cells such as Hela and 4T1 cells.

The accuracy of the sensor was evaluated with three points taken along the calibration curve (Table 1). In all cases, the sensor gave a great recovery ratio with an average of 100.76%, and the precision of the sensor was obtained with the calculated RSD of 1.86%.

### 3.5. Recyclability, Stability and Performance of The Impedimetric Sensor

**Figure 5:**
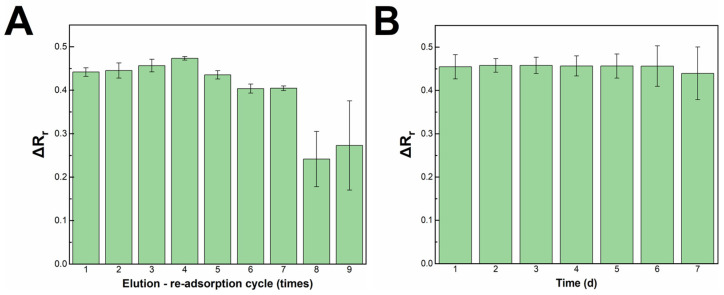
(**A**)Relative change of impedance before and after re-adsorption (in the dispersion of A549 exosomes 2.03 × 10^7^ particles/mL) obtained after the number of elution—re-adsorption cycles ranging from 1–9; (**B**) Relative change of impedance before and after re-adsorption (in the dispersion of A549 exosomes 2.03 × 10^7^ particles/mL)obtained with prepared sensors stored at 4 °C for 1–7 days (*n* = 3).

To evaluate the recyclability of the sensor, the prepared sensors were separately tested for nine cycles of elution and re-adsorption. As can be seen in Figure 5A, the response level of the sensor only changed a bit in the first seven cycles. Yet, from the 8th cycle, the response level dropped significantly, and the deviation of results measured increased considerably, which suggests the failure of the membrane structure and the disintegration of the recognition site.

Additionally, to evaluate the stability of the sensor, a set of parallel sensors was prepared and stored at 4 °C after drying under N_2_ flow. For a week, the response of the sensors was measured daily, and no significant change in response level was reported (Figure 5B). Thus, sensors prepared with this method have great stability in refrigerator.

Finally, compared to other reported methods for various kinds of exosome detection, our EIP sensor is endowed with a competitively lower LOD than all reported sensors listed in Table 2. Additionally, the few needs for antigen identification and the intrinsically label-free sensor make it an ideal tool for specific exosomes with vague morphology and immunology characters.

## 4. Conclusions

In this work, a sensitive impedimetric sensor for the A549-cells-derived exosomes based on the selectivity binding of EIP membrane and template exosomes was developed. The novel designed sensor exhibited a fast detection speed, low cost, simple operation, good recyclability and stability. Additionally, the sensor is not only sensitive to surface proteins, saccharides and glycoside markers but shape and size as well. So, it can differentiate the target exosomes from other extracellular vesicles such as microvesicles, apoptotic bodies and large oncosomes which are commonly recognized as disturbing interference in exosomes detection. With no need for antibodies, the robustness of sensors prepared as such is significantly reinforced. Moreover, the introduction of a signal label is dispensed due to the single source of impedance response caused by the adsorption of insulating exosomes. The optimization of all involved conditions grants the method with a great signal response with LOD = 2.03 × 10^3^ and LOQ = 4.10 × 10^4^ particles/mL. Furthermore, the method showed excellent accuracy and precision with a recovery ratio of 100.76% and RSD of 1.86%. The potential of this sensor was also tested for clinical translational application in culture media by taking relevant and non-relevant cells as interference, and the sensor performed well under all the conditions concerned. This experimental design can provide a novel idea for the detection of exosomes and a novel realm of MIPs application. With great competitivity for clinical translational application, this sensor is considered a novel approach to the improvement of the prognosis and survival of NSCLC patients.

## Figures and Tables

**Table 1:** Corresponding recovery ratio with evaluated accuracy and precision.

Concentration Spiked (Logarithmic Unit)	ΔR_r_	Concentration Recovered (Logarithmic Unit)	Recovery Ratio (%)	AVG (%)	RSD (%)
9.307	0.6619	9.4507	101.54	101.17	1.07
	0.6417	9.2733	99.63		
	0.6688	9.5105	102.18		
6.307	0.3339	6.5758	104.25	101.21	2.86
	0.2839	6.1378	97.31		
	0.3181	6.4372	102.06		
4.307	0.0742	4.2996	99.82	99.98	0.20
	0.0764	4.3192	100.27		
	0.0744	4.3015	99.86		
Total				100.76	1.86

**Table 2:** Performances of reported platforms for the detection of exosomes.

Substrate	Recognition Component	Detection Method	LOD (Particles/mL)	Reference
Peptides-modified gold electrodes	Peptide for EGFR/EGFRvIII and Zr-MOF for phospholipid bilayers	SWV * for MB **	7.83 × 10^6^	[28]
Aptamers-modified gold electrodes	Aptamers for CD63 and cholesterol group for phospholipid bilayers	SWV for MB	9.661 × 10^6^	[29]
Microfluidic metallic nanostructure arrays	Biotinylated anti-EpCAM	EIS	1 × 10^8^	[22]
96-well plate modified with aptamer	EpCAM aptamer and CD63 aptamer flowers	Colorimetry and photothermal	1.027 × 10^6^ for colorimetry and 2.170 × 10^6^ for photothermal	[21]
Aptamer-modified electrodes assay	EpCAM and CEA *** aptamer	Ratiometric DPV for MB and Fc ****	1.51 × 10^4^	[3]
Fe_3_O_4_ nanoparticles	MIP and CD63 aptamer	Fluorescence “turn-on”	2.43 × 10^6^	[4]
Cholesterol modified GCE	MIP membrane	EIS	2.03 × 10^3^	This work

* Squared wave voltammetry; ** Methylene blue; *** Carcinoembryonic antigen; **** Ferrocene.

## Data Availability

The data presented in this study are available on request from the corresponding author.

## Supplementary Materials

The following supporting information can be downloaded at: https://www.mdpi.com/article/10.3390/bios13060647/s1, Section S1: Methods of condition optimization; Section S2: Results and discussion of condition optimization; Figure S1: Conditional optimization of cholesteryl chloroformate; Figure S2: Conditional optimization of template exosomes; Figure S3: Conditional optimization of electro-polymerization time.
