# Supplementary material for: A Label-Free and Antibody-Free Molecularly Imprinted Polymer-Based Impedimetric Sensor for NSCLC-Cells-Derived Exosomes Detection
Source: Biosensors (Basel). 2023 Jun 13;13(6):647. doi: 10.3390/bios13060647 (PMC10296324; doi:10.3390/bios13060647)
Supplement: Supplementary file 1 [file biosensors-13-00647-s001.zip › biosensors-2396307-supplementary.pdf]

# Supplementary material

## Section S1. Methods of condition optimization

In order to obtain the optimal exosome imprinted impedance sensor, the dosage of each involved agent and treating time in each process was optimized. As to the concentration and incubation time of cholesteryl chloroformate and template A549 derived exosomes, the optimal reaction condition was determined via the change of intensity of peak current (ΔI) on SWV plot in 1× PBS (pH = 7.4) containing 10 mM $[Fe(CN)_6]^{3-/4-}$ (1:1) and 0.1 M KCl before and after the fixation of templates (in the potential window of -0.2-0.6 V). Yet for the number of cycles for electro-polymerization, elution time for template removal and re-adsorption time for sample analysis, relative difference of impedance ($\Delta R_r$) generated in the very procedure was chosen for the optimal condition in the same electrolyte solution for SWV test.

## Section S2. Results and discussion of condition optimization

Figure S1. illustrates the variation of ΔI with different concentration and treating time for the THF solution of cholesteryl chloroformate. It can be found that the concentration of 25 mM and the treating time of 30 min leads to the best current response. Larger concentration and longer time plainly cause more cholesterol group linked onto GCE, and as a result the current density is suppressed before and after incubating with template exosomes dispersion due to the insulating intrinsic of cholesterol molecule which may hinder the performance of impedance sensor via enlarging the background signal.

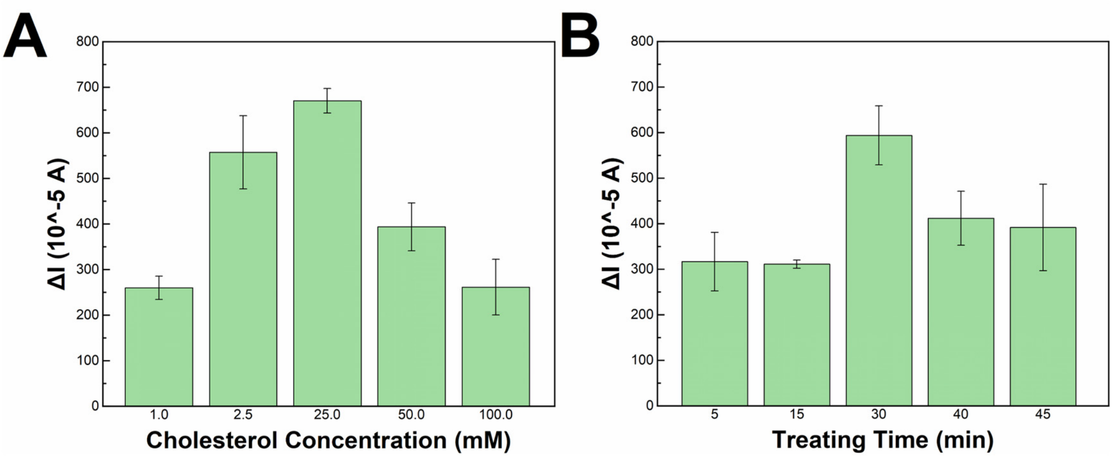


**Figure S1. Conditional optimization of cholesteryl chloroformate.**

Current response with A) concentration of cholesteryl chloroformate at 1, 2.5, 25, 50, 100 mM with treating time of 15 min. B) treating time at 5, 15, 30, 40, 45 min of cholesteryl chloroformate solution with the concentration of 25 mM (n = 3).

Furthermore, under the optimal cholesteryl chloroformate conditions, Figure S2. shows the

best current response which was afforded with the concentration of 2.03 × $10^7$ particles/mL and incubating time of 15 min. The reached plateau of ΔI when template concentration reached 2.03 × $10^7$ particles/mL implicates the saturation of template exosomes fixation, while the decrease of ΔI after 15 min is owing to the collapse of template exosomes on the GCE surface.

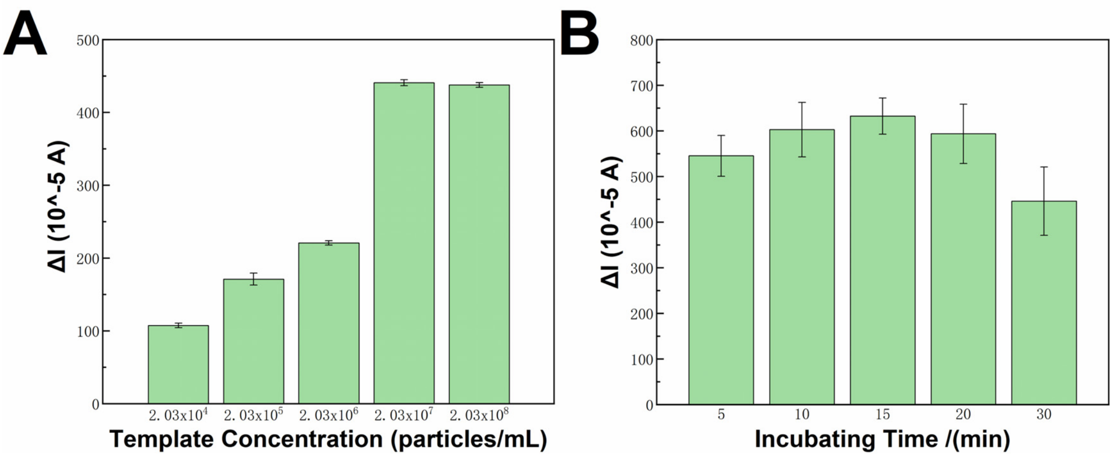


**Figure S2. Conditional optimization of template exosomes.**

Current response with A) concentration of A549 derived exosomes at 2.03 × $10^4$, 2.03 × $10^5$, 2.03 × $10^6$, 2.03 × $10^7$, 2.03 × $10^8$ particles/mL with incubating time of 10 min. B) incubating time at 5, 10, 15, 20, 30 min of A549 derived exosomes dispersion with the concentration of 2.03×$10^7$ particles/mL (n = 3).

After the optimization of process to anchor templates, the involved steps of sensor fabrication were also optimized. First, the polymerization time (a.k.a. scan cycles in electro-polymerization) was investigated. As shown in Figure S3A., membrane afforded with 10 cycles of electro-polymerization exhibited highest response of relative change of impedance. The less cycles performed, the thinner the polymer membrane grows. Therefore, the structure is vulnerable in subsequent elution process, and lower intrinsic impedance of membrane causes weaker relative change after elution. However, larger number of polymerization cycles makes the template strongly bounden to the membrane or even buried into it, so the difficulty of template removal increases in the meantime. As a consequence, 10 cycles give membranes more conducive for the templates to enter recognition sites and easier for elution.

Then the impedance response with different elution time is shown in Figure S3B. When the elution time is too short, the templates' removal is performed incompletely, which generates less recognition site available. On the other side, too long elution time damages the membrane structure to some extent, which is indicated from the larger error on response result.

Finally, it is easy to see in Figure S3C. that shorter re-adsorption time gave weaker response due to incompletely combination, while after 10 min adsorption, the saturation of re-adsorption was reached, as well as the response plateaued.

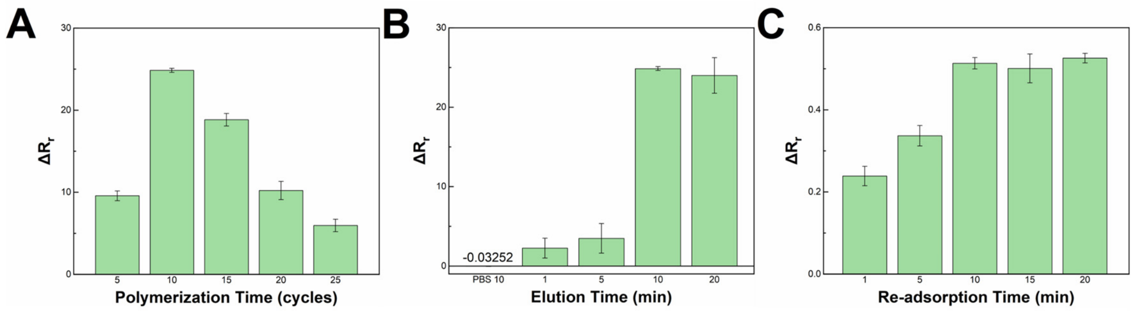


**Figure S3. Conditional optimization of electro-polymerization time.**

Impedance response of A)membrane afforded with electro-polymerization for 5, 10, 15, 20, 25 cycles before and after elution for 10 min; B)membrane afforded before and after elution with Triton X-100 in CBS for 1, 5, 10, 20 min and in PBS for 10 min; C)eluted sensors before and after re-adsorption in A549 derived exosomes dispersion with the concentration of 2.03 × $10^7$ particles/mL (n = 3).

The subsequent experiments were carried out under optimal conditions.
